# Investigating the modulation of gastric sensations and disposition toward food with taVNS

**DOI:** 10.1111/psyp.14735

**Published:** 2024-11-30

**Authors:** Andrea Salaris, Ruben T. Azevedo

**Affiliations:** ^1^ Department of Psychology Sapienza University of Rome Rome Italy; ^2^ School of Psychology University of Kent Canterbury UK

**Keywords:** appetite regulation, autonomic, gastric, interoception, vagus nerve stimulation

## Abstract

Interoception, the perception of visceral sensations, is key for several survival functions, including those related to feeding behavior. Sensations of hunger and satiety are mediated by gastric signals transmitted via the vagus nerve to the Nucleus of Solitary Tract. Transcutaneous auricular vagus nerve stimulation (taVNS) has been shown to modulate brain‐viscera communication and to impact interoceptive processing in the cardiac domain. Yet, its effect on gastric interoception remains unclear. The aim of this study was to investigate taVNS' modulatory effects on gastric interoception using the Water Load Test‐II (WLT‐II) and its impact on food‐related dispositions through a disposition and willingness to eat task (DWET). Participants underwent active or sham taVNS while performing the WLT‐II and DWET. Results showed no significant difference in gastric interoceptive accuracy and amount of water ingested between taVNS groups. However, we found a significant reduction in food liking after the fullness phase of the WLT‐II in the active (vs sham) taVNS group, suggesting an influence of vagal activation in the inhibition of food enjoyment when satiated. These findings suggest that, while taVNS may not directly enhance gastric interoceptive accuracy at a conscious level, it influences food‐related dispositions, likely by modulating the processing of gastric signals. Further research exploring the intricate relationship between vagal modulation, interoceptive abilities and eating behaviors is warranted to elucidate the underlying mechanisms and, possibly, develop targeted interventions for eating disorders.

## Abstract 2

We investigated, for the first time, if transcutaneous auricular vagus nerve stimulation (taVNS) modulates gastric interoceptive accuracy and prospective dispositions to eat in different states of stomach fullness. Although taVNS may not directly influence gastric interoceptive accuracy, we provide evidence that taVNS modulates how gastric sensations influence feeding‐related cognitions. Together these findings demonstrate the potential of taVNS as a new tool for investigating gastric‐brain communication and, eventually, the treatment of gastric‐related disorders.

## 1 INTRODUCTION

The processing and ability to sense our visceral sensations (i.e., interoception) is fundamental to maintain homeostasis in the organism and for a wide range of cognitive and affective functions (Cameron, 2001; Craig, 2009). Among visceral sensations, gastric signals are closely linked to our affective and cognitive processes. For instance, emotions such as happiness and disgust are associated with the subjective perception of changes in stomach signals (Nummenmaa et al., 2014). Recently, using an ingestible pill to record gastrointestinal physiology, research by Porciello et al. (2024) revealed that the acidity level of the stomach correlates with basic emotions: higher acidity levels are linked to increased feelings of disgust and fear, while lower acidity levels are associated with greater happiness (Porciello et al., 2024). Moreover, using the same technology Monti et al. (2022) found that physiological signals from the stomach change with different facets of bodily self‐consciousness measured throughout a virtual reality embodiment paradigm, the “Embreathment Illusion” (Cantoni et al., 2024; Monti et al., 2020, 2022).

Gastric interoceptive sensations, such as hunger and thirst are fundamental for activating and regulating feeding behavior. Indeed, research has shown that sensations of hunger and satiety are driven by our internal sensitivity to gastric signals (Herbert, 2021; Palascha et al., 2021a) and that people affected by eating disorders show altered gastric interoceptive processing and altered eating behavior (van Dyck et al., 2021; Walsh et al., 2003). Sensations of physiological hunger and satiety are facilitated by chemo‐ and mechanoreceptors located in the stomach wall stimulating vagal afferent signals from the stomach (Folgueira et al., 2014). These signals are transmitted to the nucleus of solitary tract (NTS) via the vagus nerve (Holtmann & Talley, 2014; Powley et al., 2011).

The vagus nerve (i.e., cranial nerve X) is central to the cross‐communication between the brain and the body and is a key player in conveying afferent signals from all internal organs, such as the stomach, the heart, and lungs to the NTS, a key structure for homeostatic control and energy regulation, facilitating our recognition and interpretation of physiological states (Critchley & Harrison, 2013). Moreover, efferent vagal projections modulate gastric activity, such as digestion, via the interstitial cells of Cajal (Lundgren, 1983). The existence of a gastric network in the brain and the association between cognition and stomach activity have also been convincingly demonstrated (Holtmann & Talley, 2014; Rebollo et al., 2018, 2021). Specifically, activity in a widespread cortical network is synchronized with a slow pattern of electrical activity produced by the stomach walls, known as gastric rhythm. These gastric signals are communicated to the brain via the vagus nerve and are thought to be crucial for the regulation of digestion and appetite (Browning et al., 2017; Mattes et al., 2019). Recent findings also demonstrate that the gastric rhythm is associated with other cognitive aspects such as motivation (Nord et al., 2021), reward (Neuser et al., 2020) and affect (Mayer, 2011). Interestingly, non‐invasive modulation of the auricular branch of the vagus nerve (taVNS) has been used to study this stomach‐brain interaction (Müller, Teckentrup, Rebollo, et al., 2022; Steidel et al., 2021). For example, Müller and colleagues (2022) demonstrated that vagus nerve stimulation potentiated stomach‐brain pairing activity in the NTS and enhanced the association between changes in stomach‐brain coupling and subjective ratings of hunger.

taVNS is a relatively novel and non‐invasive method to stimulate the vagus nerve, involving the application of mild electrical stimulation transcutaneously to the auricular branch of the vagus nerve located in the tragus or cymba conchae of the auricle (Peuker & Filler, 2002; Ventureyra, 2000). This technique enables the stimulation of various brain areas involved in the processing of internal bodily signals, including the brainstem and the insula (Badran et al., 2018; Toschi et al., 2023). taVNS has also been shown to modulate autonomic functioning in the cardiovascular domain, typically reflected by an increase in the vagally mediated components of heart rate variability (vmHRV) (Antonino et al., 2017; Keute et al., 2021; Machetanz et al., 2021; Toschi et al., 2023). However, it is worth noting that evidence of the effect of taVNS on HRV is mixed and the possible explanations for inconsistent findings have been recently discussed (Borges & Laborde, 2023; Wolf et al., 2021). Notably, taVNS has been employed to modulate interoceptive abilities related to perceiving heart signals (Richter et al., 2021; Villani et al., 2019). Specifically, Villani et al. (2019) showed that active taVNS improves interoceptive accuracy, as measured by the heartbeat discrimination task (Whitehead et al., 1977) and Ritcher and colleagues (2021) found that vagal nerve stimulation enhanced performance in the heartbeat counting task (Schandry, 1981). taVNS has also been found to modulate the Heart Evoked Potential (HEP), a cortical index of cardiac interoceptive processing, with a direct effect on the insula and somatosensory cortices (Poppa et al., 2022). Interestingly, Müller and colleagues (2022) also found that taVNS increases stomach‐brain coupling. Taken together these studies highlight the potential of taVNS as a tool to modulate brain–body viscera communication and, indirectly, interoception.

Surprisingly, the potential impact of taVNS in the modulation of interoceptive abilities in the gastric domain has not yet been investigated. Filling this gap in the literature could offer crucial insights into the role of the vagus nerve in the stomach‐brain connection and potentially expand the range of techniques available to treat disorders associated with interoceptive deficits, such as eating disorders or functional gastric disorders (Herbert, 2021). The principal aim of this study was to investigate the modulatory effects of taVNS in gastric interoceptive accuracy (Critchley & Garfinkel, 2017), that is, the ability to correctly identify gastric sensations, as measured with the Water Load Test‐II (WLT‐II, Van Dyck et al., 2016). The WLT‐II is a validated and widely used experimental task to study interoceptive abilities related to the stomach. This task allows us to investigate an individual's ability to perceive internal signals from the stomach and consists of drinking water until reaching subjective sensations of satiety and fullness (Van Dyck et al., 2016).

Another aim of this study was to investigate the impact of taVNS on another dimension of interoception, the preconscious impact of afferent signals on cognition (Critchley & Garfinkel, 2017). Afferent signals from the vagus nerve inform our central nervous system about the quantity (e.g., through the distension of stomach walls) and quality (e.g., nutrients) of food ingested (Wang et al., 2020), playing a key role in the regulation of food intake and subjective hunger and thirst (Müller, Teckentrup, Kühnel, et al., 2022). The mechano‐ and chemo‐sensitive properties of vagal anorexigenic fibers help transmit feedback on food intake to the brain leading to an inhibition of consumption (De Lartigue, 2016). Interestingly, vagal afferent signals have also been shown to influence mesolimbic dopamine pathways (de Araujo et al., 2012; Han et al., 2018), essential for reward processing, which, in turn, may modulate the motivational salience and hedonic value (“wanting”) of external stimuli, such as of food (Teckentrup & Kroemer, 2024). For example, Koepp et al. (2021) showed that taVNS increased “liking” ratings of food in individuals scoring high in anhedonia, a condition characterized by diminished motivation and interest to seek rewards, including food. Another study found that taVNS in combination with the consumption of a caloric milkshake increased the preference for healthy food (Öztürk et al., 2020). However, to the best of our knowledge, no study has yet investigated how taVNS influences disposition toward food as a function of stomach fullness. For this reason, the present study aimed at examining the combined effects of WLT‐II and taVNS on participants dispositions to eat, like and experience disgust for different food stimuli, as measured through a novel task adapted from the disposition and willingness to eat test (DWET, Booth, 2009; Palascha et al., 2021b). Independently of whether taVNS modulates or not the conscious perception of gastric activity and sensations of satiety (i.e., interoceptive accuracy), we may expect taVNS to impact, at a pre‐conscious level (i.e., in the absence of explicit awareness), how gastric activity and related sensations inform on the subjective appraisal of food in conditions of satiety and fullness.

We hypothesized that active (vs sham) taVNS stimulation would lead to higher scores in gastric interoceptive accuracy, as measured with the gastric sensitivity index of the WLT‐II. Moreover, in the DWET, we expected higher ratings of wanting and liking food during baseline, i.e., before starting the WLT‐II, compared to after the WLT‐II Satiety phase and the WLT‐II Fullness phase in both types of stimulation. In addition, we expected an interaction between stimulation type and water load phase, with participants reporting wanting and liking food less in the active taVNS (vs sham) at later stages of the WLT‐II. Finally, inspired by recent research showing that gastric rhythms influence sensitivity to disgust stimuli (Nord et al., 2021), we explored whether taVNS could also modulate (i.e., likely increase) reported disgust in different conditions of stomach fullness.

## 2 METHODS

### 2.1 Participants

A power analysis was conducted in MorePower 6.0.4 (Campbell & Thompson, 2012) with 80% of power and a *η* ^2^ = 0.12 (corresponding to a medium effect size) showing a minimum of 60 participants (active taVNS group: 30; sham taVNS group: 30) to detect a significant effect (*α* = 0.05). The effect size was based on a recent study by Villani and collaborators (2019) showing the effect of taVNS on cardiac interoceptive accuracy. However, a larger number of participants were recruited to compensate for possible exclusion due to failure to pass attention checks or other technical/methodological problems. We recruited 80 volunteers (7 males, M ± SD age: 19.35 ± 2.21 years), all Psychology students at the University of Kent taking part in the study in exchange for course credits. Exclusion Criteria preventing participation: (1) history of neurological disorders; (2) history of brain surgery, tumor, or intracranial metal implantation; (3) known cardiovascular abnormalities; (4) pregnancy; (5) susceptibility to seizures or migraine; (6) pacemaker or other implanted devices; (7) history of syncope; (8) particularly irritable/sensitive skin; and (9) known gastrointestinal diseases or abnormalities; (10) breach in the fasting period prior to session; (11) no alcohol/drugs taken 24 h prior to the experiment. A screening form was administered to all participants to assess their eligibility to undergo taVNS. Only volunteers who did not meet the exclusion criteria were tested. Oral and written explanations about the study were given to participants including possible adverse side effects due to taVNS stimulation (i.e., itching, burning sensations under the electrodes). All participants gave written informed consent prior to the experiment. The study was approved by the University of Kent School of Psychology Ethics committee. The pre‐registration of this study can be found here: https://aspredicted.org/Q24_H61.

### 2.2 Procedure

**FIGURE 1:**
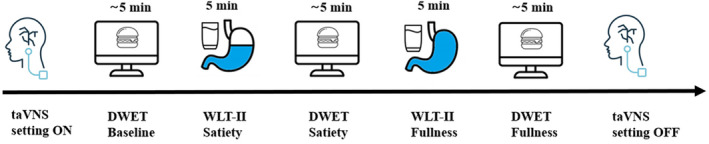
After fitting the electrodes, for active or sham taVNS, and calibrating the stimulation intensity, participants completed the DWET baseline measure with an empty stomach. Participants then completed the WLT‐II. Each phase of the WLT‐II (Satiety and Fullness) was followed by the DWET to measure changes in the appraisal of food stimuli in different conditions of stomach fullness.

Participants arrived at the lab fastened 3 h from food and 2 h from any liquids, in order to test them in conditions of relatively empty stomach, and were encouraged to use the toilet before their arrival to the lab. After reading and signing the informed consent, the electrodes for taVNS were attached and the stimulation intensity calibrated. Then, they completed the DWET at baseline, that is, with an empty stomach, followed by the first stage (Satiety) of the WLT‐II. Two additional blocks of DWET were performed, one before the second stage (Fullness) of the WLT‐II and one after it. Figure 1 shows in detail the procedure of the experiment.

### 2.3 Water load test II

The WLT‐II (Van Dyck et al., 2016) is a validated and widely used task in both clinical and experimental settings, consisting of asking participants to drink non‐carbonated water at room temperature over two successive 5‐min periods. This task comprised two phases: in the first phase, participants were instructed to drink water until they perceived the sensation of satiation (sat_ml), that is the sensation that determines meal termination. For this phase, participants were given the following instructions: “During the following five minutes, we ask you to drink water until perceiving signs of satiation. By satiation we mean the comfortable sensation you perceive when you have eaten a meal and you have eaten enough, but not too much.” In the second phase, participants were instructed to drink water until reaching the point of maximum fullness (full_ml). In this case, participants read the following instructions: “We now ask you to drink again until your stomach is completely full, that is, entirely filled with water. You have five minutes to do this”. Participants had not been informed about the second phase to avoid drinking less in the first phase. We note, however, that while we did not initially specify to participants that the task took place in two different steps, the information sheet did mention that we asked them to drink until they were satiated. Participants drank the water through long straws from non‐transparent flasks that were filled with 1.5 L of water. Participants received a refilled flask for each drinking phase, meaning that the absolute total maximum amount of water they could potentially drink was 3 L. After giving the instructions, the researcher left the room until the participant signaled that they had finished that phase. After each phase, the amount of water consumed (in milliliters) was recorded in another room using a kitchen scale (1 mL accuracy). The ratio between the volumes of water ingested in the two phases was calculated, expressing the individual index of gastric interoceptive accuracy: sat_% = sat_ml/(total_ml) × 100. The following index measures the degree to which a person is accurate in feeling signals from their stomach. The higher the index the greater the gastric interoceptive accuracy, representing an individual's subjective perception on how close satiation is to fullness, regardless of their actual stomach capacity. Other indices taken into consideration were the amount of water drunk to reach satiety (sat_ml), the amount of water drunk to reach fullness (full_ml), and the total amount of water consumed (total_ml). In addition, subjective ratings of satiety and fullness states were collected.

### 2.4 Disposition and willingness to eat task (DWET)

The disposition and willingness to eat task (DWET) was inspired by the DTE task (Booth, 2009; Palascha et al., 2021b) and was administered with E‐prime software (https://pstnet.com/products/e‐prime/). In this task, participants saw different food pictures from two different categories (savory or sweet) taken from the *food*_*pics data set* (Blechert et al., 2019). They were also shown food pictures known to elicit disgust taken from the *DIRTI* data set (Haberkamp et al., 2017). The task comprised three blocks: the *Wanting* block, in which the participant had to evaluate “how much they wanted that food now” by answering through a visual analog scale (VAS) from 0 (I don't want this food at all) to 100 (I really want this food); the *Liking* block, in which they had to evaluate “how much they liked this food now” from 0 (I don't like this food at all) to 100 (I really like this food); the *Disgust* block, in which participants had to judge “how much they felt disgusted now” on a VAS from 0 (I don't feel disgusted at all) to 100 (I am extremely disgusted). The Wanting and Liking blocks comprised 21 trials each (20 trials +1 attentional check trial taken from DIRTI data set). Both the Wanting and Liking blocks had 10 savory‐like food pictures and 10 sweet‐like food pictures (Blechert et al., 2019). The Disgust block was constituted of 20 trials (10 Disgust trials and 10 Neutral trials). Each stimulus presentation had a duration of 4 s and an interval between stimulus of 1 s. Presentations of stimuli were randomized between and within participants. The task had 62 trials in total and was repeated 3 times once before the WLT‐II and once after each WLT‐II phase (satiety and fullness). The order of the blocks was the following: Wanting, Liking, and Disgust. The entire experimental session comprised 186 trials. Table S1 of Supplementary Materials shows the number of images taken from food_pics data set and DIRTI data set.

### 2.5 Transcutaneous auricular vagus nerve stimulation

A single‐blind, sham‐controlled, between‐subjects design was used to assess the effect of taVNS on gastric interoceptive ability and ratings in the DWET. The type of stimulation (Active of Sham) was randomized between participants. Stimulation was delivered with the Transcutaneous Electrical Nerve Stimulation device (V‐TENS Plus; https://bodyclock.co.uk/). Active stimulation was achieved by passing a continuous mild electrical current, through custom‐built clip electrodes (Keute et al., 2019), into the participants' left cymba conchae, while sham stimulation was delivered in the left earlobe, which is considered to have no vagal innervations (Peuker & Filler, 2002). Stimulation, both active and sham, was delivered for the entire duration of the tasks with these parameters: pulse width = 200 μs, frequency = 24 Hz. The intensity of stimulation was set just below the participants' perceptual threshold (M ± SD active = 1.77 ± 0.73; M ± SD Sham = 1.57 ± 0.55) to avoid the activation of nociceptive fibers and possible uncomfortable and distracting sensations. This approach has been successfully used in several studies (e.g., Antonino et al., 2017; Clancy et al., 2014; Villani et al., 2019, 2022). To achieve this, the amplitude of stimulation was slowly increased until the participant reported some sensations (e.g., tingling, prickling), which was just perceived but did not cause either pain or discomfort/unpleasant sensations. The stimulation was then set to the level immediately below. The same procedure was adopted for both active and sham stimulation. At the end of the task, participants completed a questionnaire, consisting of 8 items on a Likert scale (1 = not at all; 5 = extremely) to assess sensations and adverse effects they may have felt due to the stimulation (Colzato et al., 2017).

### 2.6 Data analysis

Analyses were performed with Rstudio. Data from 10 participants were excluded due to technical problems (3) and for failing at least 3 of the 6 attention checks on DWET (7), giving a total sample of 70 participants (7 males, M ± SD age = 19.4 ± 2.32 years; active taVNS group: 35 M ± SD age = 19.8 ± 3.16 years; sham taVNS group: 35 M ± SD age = 19.1 ± 0.8 years). To determine differences between the two groups (Active stimulation vs. Sham stimulation) in the gastric interoceptive index, we used independent samples *t*‐tests when normality assumptions were met and Wilcoxon‐Mann–Whitney test when normality assumptions were violated. Tests of normality were carried out with the shapiro.test function of R. The same analysis was performed on sat_ml, full_ml, total_ml indices from the WLT‐II. Bayes Factors of the Bayesian independent two sample t‐tests were calculated for all WLT‐II indices with the BayesFactor package (Morey et al., 2015) to further support the null findings.

To test the effects of stimulation and the phases of WLT‐II on Wanting, Liking and Disgust ratings, three different linear mixed‐effects models were created, each with Wanting, Liking or Disgust VAS ratings as the dependent variable. The type of stimulation (active vs. sham), the phases of WLT‐II (baseline, satiety, and fullness), and their interaction were entered as fixed effects. The models also included subject and item (i.e., food stimuli code) as random intercepts with fixed slopes. In addition, the linear mixed‐effects model for the Disgust block also contained the Stimulus type (disgust vs. neutral) as fixed effects. Analyses were conducted in n R Studio (Allaire, 2012) with the lme4 package. *F*‐values and *p*‐values were estimated with the Anova() function from the car v3.0–3 package. Significant interactions were tested with the emmeans package (Lenth et al., 2019).

## 3 RESULTS

### 3.1 Descriptive statistics

Tables S2–S5 of Supplementary Materials show descriptive statistics.

### 3.2 Group differences in WLT‐II

Shapiro Tests revealed deviation from the normal distribution in the sat_ml of the sham group (W = 0.93, *p*‐value = .02), full_ml of the sham (*W* = 0.93, *p*‐value = .02), and active group (*W* = 0.93, *p*‐value = .02). Considering the violation of normality assumption for sat_ml and full_ml, the Wilcoxon rank‐sum test was used for these variables, while student's test for the others.

**FIGURE 2:**
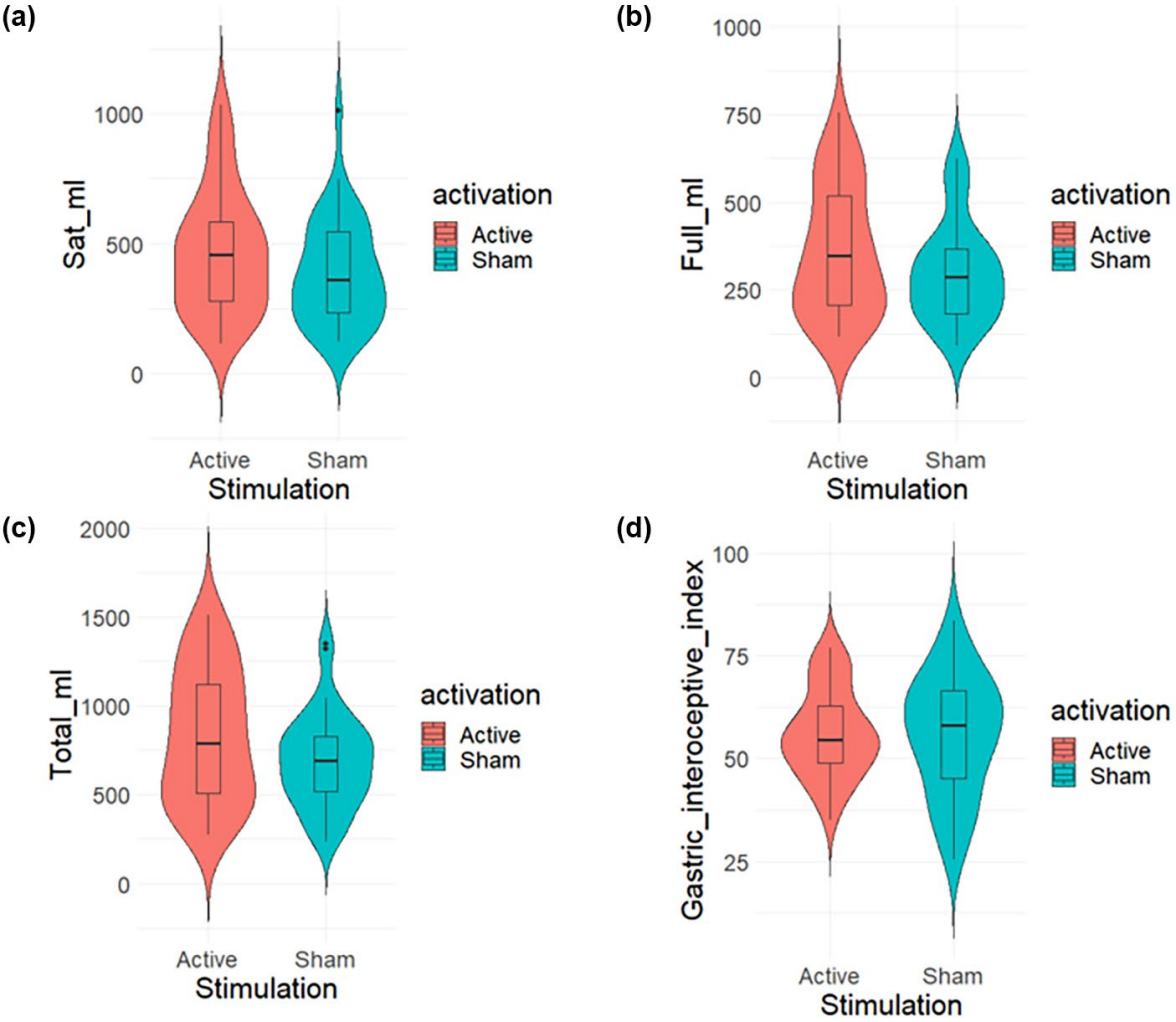
The different WLT‐II indices for each group: (a) The amount of water drunk to reach perceived satiety (Sat_ml) and (b) Fullness (Full_ml); (c) The total amount of water consumed (Total_ml); (d) Gastric interoceptive accuracy index.

The independent two‐ samples *T*‐test did not reveal any significant difference between groups in the gastric interoceptive accuracy index (*t* (62.65) = 0.16, *p*‐value = .87, BF_10_ = 0.24; M ± SD in group Active = 56.25 ± 10.8; M ± SD in group Sham = 55.76 ± 14.6). Considering the total amount of water consumed, no difference emerged between the active and sham stimulation (*t* (61.05) = 1.8063, *p*‐value = .07, BF_10_ = 0.97; M ± SD in the Active group = 830.26 ± 347.7; M ± SD in the Sham group = 690.34 ± 263.8). Analyses on each WLT‐II phase did not reveal any consistent difference between groups in the amount of water drunk to reach satiety (*W* = 505.5, *p*‐value = .19; BF_10_ = 0.62, M ± SD active group = 468.88 ± 237.2; *M ± SD* sham group = 391.40 ± 201.2) nor to reach fullness (*W* = 504.5, *p*‐value = .21, BF_10_ = 0.71; M ± SD active group = 361.37 ± 185.7; M ± SD sham group = 298.94 ± 141.2). In addition, we re‐run these analyses after excluding data from behavioral outliers (identified with the interquartile range technique, *n* = 10) and found equivalent results. Figure 2 illustrates the gastric interoceptive index and water consumption in the two groups.

### 3.3 Linear mixed‐effect models in DWET

#### 3.3.1 Liking

**FIGURE 3:**
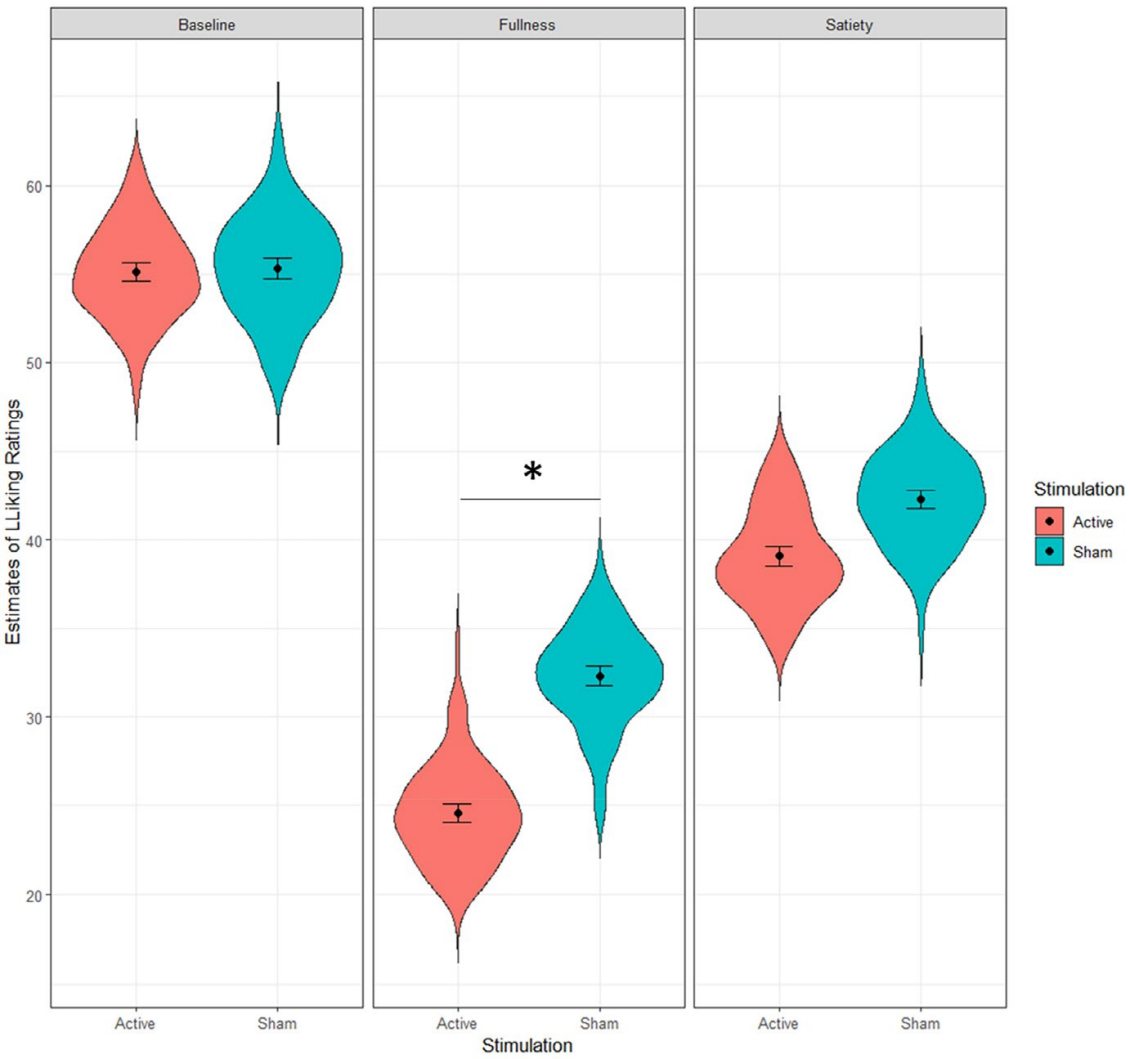
Significant interaction between WLT‐Session and type of taVNS stimulation. The plot shows the fixed effect estimates extracted from the mixed model and describes a significant less liking of food in the fullness phase in participants who underwent taVNS compared to those receiving sham stimulation.

The model on the liking of food revealed a significant main effect of WLT‐II phase (*F*[2,4107] = 354.42, *p* < .001) and no significant effect of stimulation type (*F*[1,68] = 1.44, *p* = .23). Interestingly, a significant interaction between type of stimulation and water load phases emerged (*F*[2,4107] = 5.32, *p* = .004). FDR‐corrected post hoc analyses revealed a significant difference between groups in the fullness phase (*z*‐ratio = −2.156, *p* = .03) but not in the other phases (*ps* > .05). Gastric sensations induced by the WLT‐II were effective in changing subjective appraisal (i.e., liking) of food, especially for participants receiving transcutaneous vagus nerve stimulation. Figure 3 shows the significant interaction between WLT‐II phase and stimulation type.

To confirm that the results observed in the post hoc analysis were effectively explained by a greater decrease in Liking after water consumption in the active (vs sham) group, and not by between‐group differences already present at baseline, an index of Δ Liking ratings was estimated by dividing ratings from the fullness phase with those from the baseline phase. An independent sample *t*‐test confirmed a significant difference between groups (*t* (68) = −2.1345, *p*‐value = .03, M ± SD in active group = 0.44 ± 0.27, M ± SD in sham group = 0.58 ± 0.27).

#### 3.3.2 Wanting

**FIGURE 4:**
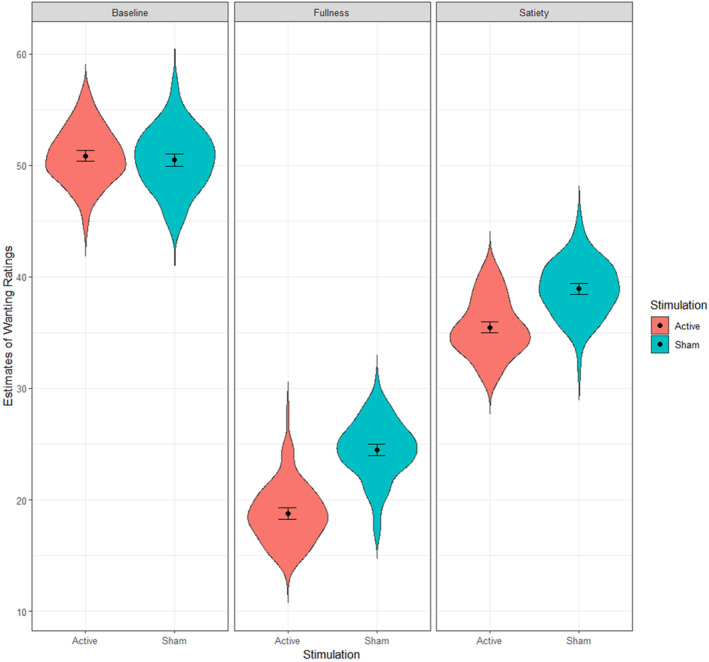
Significant interaction between WLT‐Session and type of taVNS stimulation in Wanting ratings. The plot shows the fixed effect estimates extracted from the mixed model.

The linear mixed model on the wanting of food showed a significant main effect of WLT‐II phase (*F*[2,4107] = 518.77, *p* < .001), revealing a decrease in participants' appetite for food after reaching sensations of satiety and fullness. No significant effect was found for the type of stimulation (*F*[1,68] = 0.82, *p* = .33). Conversely, results did show a significant interaction between type of stimulation and WLT‐II phase (*F*[2,4107] = 4.85, *p* = .007), but FDR‐corrected post hoc analyses did not reveal any significant difference between groups in neither of the WLT‐II phases (active vs. sham baseline: *z*‐ratio = 0.014, *p* = .98; active versus sham satiety: *z*‐ratio = −1.114, *p* = .28; active versus sham fullness = *z*‐ratio = −1.509, *p* = .15). Figure 4 shows the significant interaction in Wanting ratings between stimulation and WLT‐II phases.

#### 3.3.3 Disgust

The model investigating the modulation of disgust through taVNS, and WLT‐II showed significant main effects of WLT‐II phases (*F*[2,4107] = 11.61, *p* < .001) and type of stimuli (*F*[1,18] = 809.96, *p* < .001) but no main effect of stimulation (*F*[1,69] = 0.56, *p* = .45) and no interaction between factors was found to be significant (*ps* > .05). These results reveal that WLT‐II increased general feelings of disgust toward food but that this was not modulated by taVNS.

## 4 DISCUSSION

There is growing evidence on the influence of taVNS on gastric activity (Du et al., 2024; Hong et al., 2019; Steidel et al., 2021; Teckentrup et al., 2020) and stomach‐brain interaction (Müller, Teckentrup, Rebollo, et al., 2022). For example, research has found that this type of non‐invasive stimulation produces changes in gastric frequency and digestive activity through the activation of the dorsal vagal complex (Teckentrup et al., 2020); can increase gastroduodenal motility (Frøkjaer et al., 2016); is able to alter gastric motility by causing higher amplitudes of peristaltic waves (Steidel et al., 2021); and normalize gastric dysrhythmias (Du et al., 2024). To the best of our knowledge, this study is the first to investigate whether taVNS modulates the conscious perception of gastric sensations of fullness, as measured through the WLT‐II, and if such sensations influence the subjective appraisal of food. While we found no evidence for an effect of vagus nerve stimulation on the WLT‐II's index of gastric interoceptive accuracy, our results revealed that taVNS modulates the subjective appraisal of food in conditions of stomach fullness.

Contrary to our expectations, taVNS did not modulate the gastric interoceptive accuracy index. There may be different explanations for this result. Firstly, while the WLT‐II is, undoubtedly, the most widely used task to measure gastric interoception, it does have several limitations. For example, it has been argued that it can be influenced by different variables such as guessing strategies, response bias, stomach capacity, thirst, and internal beliefs or attitudes (e.g., fear of drinking too much water) (Desmedt et al., 2023; Van Dyck et al., 2016). Nonetheless, even if currently this is the only non‐invasive gastric interoceptive task, it is possible that this task is not sensitive enough to measure modulations of interoceptive accuracy, particularly when using between‐subject designs that are more prone to such inter‐individual confounds. Indeed, in the cardiac domain, taVNS was found to enhance interoceptive accuracy in the heartbeat discrimination task but not in the heartbeat tracking task, a task known to be more susceptible to confounds and subjective biases (Villani et al., 2019). We also note that research has provided mixed findings on the relationship between interoceptive abilities in these two sensory modalities. While an association between gastric and cardiac channels was observed by Herbert and collaborators (Herbert et al., 2012), where individuals who were better at perceiving their heartbeats were also more sensitive to gastric signals, a more recent study failed to confirm this relationship between the gastric and cardiac axes (Ferentzi et al., 2018). Thus, it may also be the case that taVNS does not modulate explicit awareness of gastric sensations of fullness. Despite the known role of the vagus nerve in the regulation and feedback to the brain of gastric activity (Browning et al., 2017; Müller, Teckentrup, Rebollo, et al., 2022), it is possible that the vagus does not play a direct role in bringing gastric sensations to conscious awareness. However, we argue that it is more likely that experimental limitations, rather than the lack of modulatory potential, are at the base of our null findings. Not only taVNS has been shown to increase stomach‐brain coupling and modulate brain activity in cortical regions associated with changes in hunger ratings, suggesting an influence on gastric interoceptive processing (Müller, Teckentrup, Rebollo, et al., 2022), as our results show a taVNS influence on how sensations of fullness influence the appraisal of food.

We also did not find any effect of taVNS on drinking behavior, that is, on the amount of water consumed during the WLT‐II. Interestingly, however, we found a significant interaction between the WLT‐II phase and the type of stimulation in the modulation of dispositions toward food. Specifically, our results suggest that vagus nerve stimulation decreases the prospective liking of food when the stomach is full, highlighting the potential of taVNS to modulate, at a pre‐conscious level, how interoceptive signals impact cognition. taVNS was previously shown to influence gastric motility. For example, Steidel et al. (2021) demonstrated that high‐frequency (25 Hz) taVNS led to an increase in gastric motility, compared to low‐intensity (1 Hz) taVNS. The authors also found significantly higher amplitudes of peristaltic waves due to taVNS. Gastric motility is known to be a key mediator for hunger and satiety (Janssen et al., 2011). Both higher peristaltic waves and the enhancement of gastric motility stimulate mechanoreceptors and chemoreceptors in the stomach, enhancing afferent traffic to the brain and, consequently, promote stronger perceptions of fullness and other gastric sensations (Janssen et al., 2011; Li & Page, 2022; Mercado‐Perez & Beyder, 2022; Shahsavari & Parkman, 2022). Thus, it is likely that taVNS modulated the afferent vagal traffic and/or the strength of the representations of these signals of gastric fullness which in turn reduced the hedonic responses toward food.

This result corroborates previous studies demonstrating that a chronic administration of direct cervical vagus nerve stimulation promotes the regulation of eating behavior and subsequent weight loss (Burneo et al., 2002; Pardo et al., 2007). Invasive vagus nerve stimulation has demonstrated to be effective in limiting food intake in animals (Dai et al., 2020), even if evidence in humans is still mixed (e.g., Pelot & Grill, 2018). Nonetheless, some studies suggest that taVNS has an effect in normalizing hedonic response toward food (e.g., liking) in patients with depression, by increasing linking ratings of food when anhedonia is present (Koepp et al., 2021). The results of the latter study are particularly interesting for the present discussion as, both the reported increased liking of food in conditions of hunger and our findings of decreased liking of food in conditions of fullness, suggest that taVNS may increase body‐to‐brain communication to regulate hunger/satiety states.

These results, however, are in apparent contrast with recent studies showing either no modulation or contrasting effects of taVNS on eating dispositions (Müller, Teckentrup, Kühnel, et al., 2022; Öztürk et al., 2020), In particular, Müller and colleaguesec (2022) did not find changes in self‐reported hunger or thirst after taVNS (vs. sham) and Öztürk and colleagues (2020) reported, in a pilot study (*n* = 10), increased liking of low‐ (but not high‐) fat food after taVNS. However, in these studies, participants were either not asked to ingest any food or liquids (Müller, Teckentrup, Kühnel, et al., 2022) or researchers did not measure how the subjective appraisal of food changes after the ingestion of food or liquids (Öztürk et al., 2020). Interestingly, recent research by Altinkaya and colleagues (2023) used taVNS in different locations (cymba conchae, tragus, or both) to investigate if vagal stimulation modulated the appraisal of palatable drinks (by measuring “Wanting” and “Liking” ratings) and physiological variables (HRV and gastric frequency), before and after drinking a chocolate flavored milk. The authors found that stimulation in cymba conchae was associated with a decrease in wanting ratings after ingesting the palatable drink (Altınkaya et al., 2023). In the present study, we show that taVNS modulates how feeding‐related dispositions change in different conditions of stomach fullness due to the ingestion of water, that is, a non‐caloric and “tasteless” drink. Thus, unlike previous studies in which the ingestion of caloric/palatable drinks is likely to trigger other neuroendocrine hunger regulation mechanisms sensitive to taste and nutrient content, our study taps more directly into the effects of taVNS due to the distension of the stomach walls.

Indeed, there is a substantial body of literature showing that the vagus nerve helps signal information not only about the properties of a meal, such as its nutrients, but also about the quantity of food and liquids ingested. The latter is achieved through mechanosensors detecting volume changes in the upper gastrointestinal tract, which at a central level are translated into appetite‐suppressing signals (Browning et al., 2017; Kaniusas et al., 2019; Mercado‐Perez & Beyder, 2022; Wang et al., 2020). The modulation of hunger and thirst‐regulating neural systems (e.g., brainstem; hypothalamus; insula) is also likely to change the motivational and operant responses to visual cues of food (Décarie‐Spain et al., 2024). This could explain our result of reduced prospective liking of food in conditions of full stomach during active taVNS (vs. sham) Future studies should induce satiety/fullness states in participants after the ingestion of a caloric drink to investigate how taVNS modulates the appraisal of food through these two different mechanisms sensing the quality or quantity of a meal.

The use of WLT‐II to investigate gastric interoceptive accuracy can be seen as a limitation of the present study. Newer tasks to explore the ability to read gastric sensations have emerged in recent years. For example, in the glucose test participants have to report their sensations of satiety while glucose levels in the blood are continuously monitored (Young et al., 2021). In the gastrointestinal mechanosensory stimulation task participants ingest a vibrating capsule and have to tap a button each time they feel vibration in their stomach (Smith et al., 2021). Despite the promising value of these two tasks, feasibility considerations (e.g., cost and setting) represent a great obstacle to their use (Desmedt et al., 2023). Also, while our sample size was determined a priori through power analyses, it may be argued that the study lacks some statistical power that prevents the confident interpretation of statistical trends (i.e., effect of taVNS on amount of water ingested) and lack of significance in some post hoc analyses (i.e., in the Wanting ratings). Finally, it would be interesting to replicate this study: (i) using different stimulation parameters (see for example, D'Agostini et al., 2023; Villani et al., 2022) (ii) after the ingestion of different foods/liquids; (iii) and with different timelines between the food/liquid ingestion and the DWET, to test the effects of taVNS on dispositions to eat at different stages of digestion (e.g., Gonzalez‐Izundegui et al., 2021; Goyal et al., 2019). Another limitation of the present study is the lack of a blinding check that would have allowed to exclude possible expectancy confounds, even considering that participants were naïve to the specificities of taVNS and that both stimulations (sham or active) induce similar sensations (e.g., tingling). Lastly, a further limitation is the non‐use of a cross‐over design and the lack of investigation of group differences before the stimulation.

In sum, we did not find evidence that taVNS modulates gastric interoceptive accuracy. However, we observed a tendency to like food pictures less during states of gastric fullness after active (vs. sham) taVNS. Together, these results demonstrate how taVNS influences the gut‐to‐brain axis by modulating how gastric sensations influence behavior and feeding‐related cognition. This study also highlights how this novel task (DWET) can be used in combination with the Water Load Test‐II as an experimental manipulation to investigate how feelings of satiety modulate subjective states related to food. Future research could expand these results by further exploring the role of the vagus nerve in gastric interoception, for example, using a different gastric interoceptive ability task, changing stimulation parameters, or the timeline between the ingestion of food/liquids and measures of dispositions to eat. Considering the promising results of taVNS on the inhibition of food intake (Dai et al., 2020), stabilization of hedonic responses toward food (Koepp et al., 2021; Öztürk et al., 2020), and treatment of psychiatric symptomatology underlying eating disorders (Gallop et al., 2022; Melis et al., 2020), future research should evaluate the integration of taVNS in psychological and pharmacological treatments of eating disorders (e.g., binge eating disorders or obesity).

## AUTHOR CONTRIBUTIONS

**Andrea Salaris:** Conceptualization; data curation; formal analysis; investigation; methodology; project administration; visualization; writing – original draft; writing – review and editing. **Ruben T. Azevedo:** Conceptualization; formal analysis; methodology; resources; software; supervision; validation; visualization; writing – review and editing.

## FUNDING INFORMATION

This work was supported also by “Finanziamento dell'Unione Europea ‐ NextGenerationEU‐ missione 4, componente 2, investimento 1.1.” PRIN PNRR (Project Code: P2022TPX8E ‐CUP: B53D23030340001 ‐CUP MASTER: B53D23030340001) to A.S.

## CONFLICT OF INTEREST STATEMENT

The authors declare no conflict of interest.

## Supporting information


**Table S1:** Pictures of DWET for Wanting, Liking and Disgust blocks (Haberkamp et al., 2017; Blechert et al., 2019).
**Table S2:** Mean and SD (standard deviation) of Age, MIRES questionnaire and MAIA subscales for each taVNS group (active vs. sham).
**Table S3:** Mean and SD of Wanting and Liking scores for each taVNS group (active and sham) and WLT‐phases (baseline, satiety and fullness).
**Table S4:** Mean and SD of Disgust scores based on the type of stimuli for each taVNS group (active and sham) and WLT‐phases (baseline, satiety and fullness).
**Table S5:** Mean and SD of self‐report ratings of perceived Satiety and Fullness in each WLT‐phases (baseline, satiety and fullness).

## Data Availability

The data sets generated and analyzed during the current study are available on the Open Science Framework repository https://osf.io/m8cyf/?view_only=2ed5744198cd42cdab95ce5dbb527b14.
