# Supplementary material for: Investigating the modulation of gastric sensations and disposition toward food with taVNS
Source: Psychophysiology. 2024 Nov 30;62(2):e14735. doi: 10.1111/psyp.14735 (PMC11870815; doi:10.1111/psyp.14735)
Supplement: Supplementary file 1 — Table S1: Pictures of DWET for Wanting, Liking and Disgust blocks (Haberkamp et al., 2017; Blechert et al., 2019). Table S2: Mean and SD (standard deviation) of Age, MIRES questionnaire and MAIA subscales for each taVNS group (active vs. sham). Table S3: Mean and SD of Wanting and Liking scores for each taVNS group (active and sham) and WLT‐phases (baseline, satiety and fullness). Table S4: Mean and SD of Disgust scores based on the type of stimuli for each taVNS group (active and sham) and WLT‐phases (baseline, satiety and fullness). Table S5: Mean and SD of self‐report ratings of perceived Satiety and Fullness in each WLT‐phases (baseline, satiety and fullness). [file PSYP-62-e14735-s001.docx]

**SUPPLEMENTARY MATERIALS**

**DWET’s stimuli**

DWET was developed throughout software (Psychology Software Tools, Inc.).

Stimuli were chosen from food_pic Dataset (Blechert et al., 2019) and from DIRTI Dataset food category (Haberkamp et al., 2017).

| **Wanting** | **Liking** | **Disgust** |
| --- | --- | --- |
| 593 | 140 | 1024_food |
| 337 | 86 | 1046_food_neutral |
| 4 | 238 | 1047_food_neutral |
| 27 | 705 | 1048_food_neutral |
| 65 | 287 | 1007_food |
| 1 | 715 | 1014_food |
| 307 | 82 | 1024_food |
| 131 | 391 | 1010_food |
| 492 | 568 | 1009_food |
| 378 | 13 | 1006_food |
| 392 | 73 | 1045_food_neutral |
| 682 | 761 | 1004_food |
| 483 | 792 | 1003_food |
| 452 | 567 | 1044_food_neutral |
| 188 | 560 | 1041_food_neutral |
| 551 | 749 | 1043_food_neutral |
| 90 | 313 | 1049_food_neutral |
| 107 | 555 | 1042_food_neutral |
| 56 | 357 | 1050_food_neutral |
| 491 | 117 | 1015_food |

***Table S1*** *Pictures of DWET for Wanting, Liking and Disgust blocks (Haberkamp et al., 2017; Blechert et al., 2019).*

|  | | | | | | | |
| --- | --- | --- | --- | --- | --- | --- | --- |
|  | | **taVNS** | | **Mean** | | **SD** | |
| Age |  | Active |  | 19.80 |  | 3.160 |  |
|  |  | Sham |  | 19.06 |  | 0.802 |  |
| MIRES_TOT |  | Active |  | 102.23 |  | 25.735 |  |
|  |  | Sham |  | 107.89 |  | 20.458 |  |
| Trusting |  | Active |  | 3.34 |  | 1.071 |  |
|  |  | Sham |  | 3.65 |  | 0.808 |  |
| Body_Listening |  | Active |  | 3.06 |  | 0.962 |  |
|  |  | Sham |  | 3.13 |  | 0.751 |  |
| Self_Regulation |  | Active |  | 2.95 |  | 0.778 |  |
|  |  | Sham |  | 3.22 |  | 0.603 |  |
| Emotional_Awareness |  | Active |  | 3.75 |  | 0.832 |  |
|  |  | Sham |  | 3.75 |  | 0.620 |  |
| Attentional_Regulation |  | Active |  | 3.21 |  | 0.660 |  |
|  |  | Sham |  | 3.25 |  | 0.495 |  |
| Not_Worrying |  | Active |  | 2.27 |  | 0.797 |  |
|  |  | Sham |  | 2.18 |  | 0.642 |  |
| Not_Distracting |  | Active |  | 1.57 |  | 0.817 |  |
|  |  | Sham |  | 1.51 |  | 0.684 |  |
| Noticing |  | Active |  | 3.66 |  | 0.610 |  |
|  |  | Sham |  | 3.86 |  | 0.455 |  |
|  | | | | | | | |

***Table S2*** *Mean and SD (standard deviation) of Age, MIRES questionnaire and MAIA subscales for each taVNS group (active vs sham)*

|  | | | | | | | | | |
| --- | --- | --- | --- | --- | --- | --- | --- | --- | --- |
|  | | **WLT-II phase** | | **taVNS** | | **Mean** | | **SD** | |
| Wanting_VAS |  | Baseline |  | Active |  | 50.6 |  | 30.6 |  |
|  |  |  |  | Sham |  | 50.6 |  | 31.4 |  |
|  |  | Fullness |  | Active |  | 19.1 |  | 22.0 |  |
|  |  |  |  | Sham |  | 24.4 |  | 24.0 |  |
|  |  | Satiety |  | Active |  | 35.1 |  | 28.7 |  |
|  |  |  |  | Sham |  | 39.1 |  | 29.1 |  |
| Liking_VAS |  | Baseline |  | Active |  | 54.8 |  | 32.0 |  |
|  |  |  |  | Sham |  | 55.1 |  | 33.4 |  |
|  |  | Fullness |  | Active |  | 24.9 |  | 25.2 |  |
|  |  |  |  | Sham |  | 31.7 |  | 28.5 |  |
|  |  | Satiety |  | Active |  | 38.7 |  | 30.0 |  |
|  |  |  |  | Sham |  | 42.2 |  | 30.9 |  |
|  | | | | | | | | | |

***Table S3*** *Mean and SD of Wanting and Liking scores for each taVNS group (active and sham) and WLT-phases (baseline, satiety and fullness)*

|  | | | | | | | | | | | |
| --- | --- | --- | --- | --- | --- | --- | --- | --- | --- | --- | --- |
|  | | **WLT-II phase** | | **Stimuli** | | **taVNS** | | **Mean** | | **SD** | |
| Disgust_VAS |  | Baseline |  | Disgust |  | Active |  | 89.7 |  | 19.5 |  |
|  |  |  |  |  |  | Sham |  | 90.5 |  | 20.1 |  |
|  |  |  |  | Neutral |  | Active |  | 15.8 |  | 22.8 |  |
|  |  |  |  |  |  | Sham |  | 18.7 |  | 24.0 |  |
|  |  | Fullness |  | Disgust |  | Active |  | 92.2 |  | 16.6 |  |
|  |  |  |  |  |  | Sham |  | 93.7 |  | 13.9 |  |
|  |  |  |  | Neutral |  | Active |  | 21.3 |  | 25.8 |  |
|  |  |  |  |  |  | Sham |  | 21.0 |  | 23.9 |  |
|  |  | Satiety |  | Disgust |  | Active |  | 91.4 |  | 17.9 |  |
|  |  |  |  |  |  | Sham |  | 93.7 |  | 13.1 |  |
|  |  |  |  | Neutral |  | Active |  | 17.9 |  | 24.2 |  |
|  |  |  |  |  |  | Sham |  | 19.0 |  | 23.0 |  |
|  | | | | | | | | | | | |

***Table S4*** *Mean and SD of Disgust scores basing of type of stimuli for each taVNS group (active and sham) and WLT-phases (baseline, satiety and fullness)*

|  | | **WLT-II phase** | | **Mean** | | | **SD** | | |  |
| --- | --- | --- | --- | --- | --- | --- | --- | --- | --- | --- |
| Satiety |  | Baseline |  |  | 2.16 |  | | 1.16 |  | |
|  |  | Fullness |  |  | 5.86 |  | | 1.66 |  | |
|  |  | Satiety |  |  | 5.61 |  | | 1.09 |  | |
| Fullness |  | Baseline |  |  | 1.79 |  | | 1.06 |  | |
|  |  | Fullness |  |  | 6.59 |  | | 0.67 |  | |
|  |  | Satiety |  |  | 5.43 |  | | 1.15 |  | |

***Table S5*** *Mean and SD of self-report ratings of perceived Satiety and Fullness in each WLT-phases (baseline, satiety and fullness)*
